# [Retracted] Clinical significance and effect of Sam68 expression in gastric cancer

**DOI:** 10.3892/ol.2025.15416

**Published:** 2025-12-02

**Authors:** Jinzhang Xiao, Qiuhong Wang, Qichang Yang, Hua Wang, Fulin Qiang, Song He, Jin Cai, Lei Yang, Yingying Wang

Oncol Lett 15: 4745–4752, 2018; DOI: 10.3892/ol.2018.7930

Following the publication of the above paper, a concerned reader drew to the Editor's attention that the GAPDH control western blots shown in Fig. 1A (lower panel) on p. 4747 and the scratch-wound assay images shown in Fig. 4A on p. 4751 had apparently already appeared in previous papers written by different authors at different research insitutes in the journals *PLoS One* and *Molecular Biology of the Cell*, the first of which has since been retracted.

Given that the abovementioned data had already been published previously prior to the submission of this paper to *Oncology Letters*, the Editor has decided that this article should be retracted from the Journal. The authors were asked for an explanation to account for these concerns, but the Editorial Office did not receive a reply. The Editor apologizes to the readership for any inconvenience caused.

